# Delta thermal radiomics: An application in dairy cow teats

**DOI:** 10.3168/jdsc.2021-0179

**Published:** 2022-02-10

**Authors:** P.S. Basran, C. DiLeo, Y. Zhang, I.R. Porter, M. Wieland

**Affiliations:** 1 Department of Clinical Sciences, College of Veterinary Medicine, Cornell University, Ithaca, NY 14853; 2 College of Veterinary Medicine, Cornell University, Ithaca, NY 14853; 3 Department of Population Medicine and Diagnostic Sciences, College of Veterinary Medicine, Cornell University, Ithaca, NY 14853

## Abstract

We describe a novel approach for analyzing thermal images by way of radiomics (i.e., thermal radiomics) and how it can be used to monitor short-term temperature changes of dairy cow hind teats; that is, delta thermal radiomics. The heat generated from metabolic activities and blood-flow patterns can be visualized using thermal radiography of the skin surface. The hind teats from 25 dairy cows were imaged with a digital thermal camera and the images were converted to medical images (DICOM format) by mapping the multi-channel colorized thermal image to a monochromatic image whose intensities represent temperature. The 50 teats (left and right hind) were then manually segmented by 2 investigators. Radiomics analysis, which is a common method of extracting semantic and nonsemantic image biomarkers from medical images for machine learning, was performed. To evaluate whether this approach can detect pre- and postmilking differences, 18 cows were imaged before and after milking, the teats were manually segmented, and radiomic calculations were performed. Student's *t*-test was used to provide an estimate of the likelihood of whether postmilking thermal image biomarkers are the same as premilking thermal image biomarkers, and Cohen's *d* was used to evaluate the size of the effect (*d* > 1.2). To evaluate uncertainties from manual segmentation, the Dice similarity score (DS) between the 2 investigators' segments was computed. The average DS (95% confidence limit) was 0.952 (0.913–0.982) when comparing the 2 investigators' segmentations. There was no significant difference in DS when comparing the left and right segmented teats, suggesting that teats can be segmented consistently. No differences (*d* < 0.36) were observed when comparing image biomarkers from one investigator's segments with the other's, suggesting that image biomarkers computed from one investigator's segmentation of teats are not likely to differ from those computed from the other investigator. When comparing image biomarkers before and after milking, 109 image biomarkers were analyzed, and 17 image biomarkers were simultaneously significant and exhibited effect size. Thus, delta thermal radiomics offers a noninvasive and quantitative method of monitoring skin temperature changes in humans and animals after an intervention. The advantage of this approach is that it can reveal both perceptible and imperceptible surface temperature features that may be useful for detecting and managing dairy teat health.

## Highlights

• Infrared thermograms can indirectly detect blood flow and changes in blood flow on the skin.
• Radiomics is a machine learning medical image analysis technique that reveals semantic and nonsemantic features.
• Radiomics of dairy cow teat thermograms is a novel quantitative means of assessing changes in skin temperature before and after milking.

## Graphical Abstract

**Figure:**
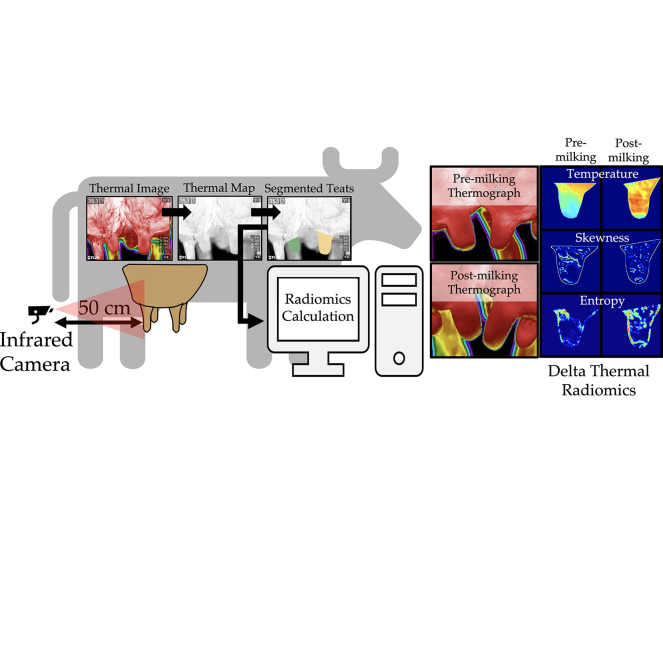
**Summary:** In the delta thermal radiomics workflow, infrared thermal images of the hind teats of dairy cows are collected and the 3-channel color images are converted to a single-channel medical image. The medical image is then read using open-source medical image analysis software and the image biomarkers (radiomics) are computed over the segmented hind teats. Expressed delta radiomic features (pre- and postmilking) may be visualized with feature maps. Shown here are feature maps that exhibit both statistical differences (Student's *t*-test, P < 0.01) and a strong effect size (Cohen's *d* > 1.2).

Radiomics borrows the “omics” suffix from other emerging fields of study, such as genomics, proteomics, and metabolomics, where image biomarkers are extracted from a 2-dimensional or 3-dimensional (**3D**) image rather than a biospecimen (Gillies et al., 2016). The idea underlying radiomics is that quantitative image features, or image biomarkers, can be identified from a region of interest in a medical image, similar to how a complete blood count or cell types can be identified from a blood sample or biopsy. The motivation for computing these image biomarkers stems from the idea that the underlying pathophysiology, or responses to treatment, can be detected through changes or differences in image biomarkers, and that expressed image biomarkers could be used in classification or regression modeling (Lambin et al., 2012; Aerts et al., 2014). Radiomics can also reveal patterns and texture features that cannot be easily perceived by the human eye. Using open-source and easily accessible image analysis and software packages, radiomics analysis has been applied in several medical applications, including detecting patients with COVID-19 from chest X-rays, predicting responses to cancer therapies, and identifying pathologic features in the bones of racehorses that have suffered catastrophic injuries (Horvat et al., 2018; Basran et al., 2021; Xiao et al., 2021). Radiomics analysis follows several distinct steps: image acquisition and data preparation, segmentation, and feature calculation. After feature selection, image biomarkers can be used in modeling. Features can be categorized by the region of interest's shape (e.g., area, perimeter, elongation), and first-order statistics of the intensities inside the region of interest (e.g., mean, standard deviation, kurtosis). Complex texture features of the image intensities can also be extracted. These higher-order features provide insight into the spatial arrangement of intensities, similarities in intensities along vertical, horizontal, and diagonal directions, and pixels with connected and similar intensities (van Griethuysen et al., 2017; Basran et al., 2021). These texture features are often computed by representing image intensities (e.g., pixels of temperature) in the region of interest (e.g., left or right hind teats) as a matrix from which an image biomarker can be extracted. A more recent trend of radiomics studies is monitoring the change in image features—that is, delta radiomics—after a patient undergoes an intervention, such as predicting the outcomes for lung cancer patients (Fave et al., 2017). To our knowledge, radiomics analysis has not been explored in the dairy industry.

Thermal radiography provides a 2-dimensional image consisting of pixel intensities of temperature distributions, often expressed through a multi-channel colorized image (Figure 1). The temperature changes from metabolic activities and blood-flow patterns can be visualized with thermal radiography of the skin surface. In the dairy industry, thermal radiography has been used to monitor udder health and mastitis (Paulrud et al., 2005; Colak et al., 2008; Velasco-Bolaños et al., 2021). Preclinical blood-flow patterns can be detected with thermal radiography, in potential applications such as monitoring breast cancers (Gautherie and Gros, 1980). In the mid-2010s, excitement in the potential use of thermal imaging as a noninvasive mammography screening tool waned because of the extent and need for manual expertise in interpreting breast thermograms. However, machine-learning approaches offer an opportunity to reduce the extent of and need for manual expertise. The concept of using radiomics-based thermal image biomarkers and machine learning as a noninvasive tool for breast cancer screening has recently been investigated (Kakileti et al., 2020). In this approach, thermal images are quantitatively analyzed to objectively score the risk of breast cancer through a machine-learning approach.

**Figure 1:**
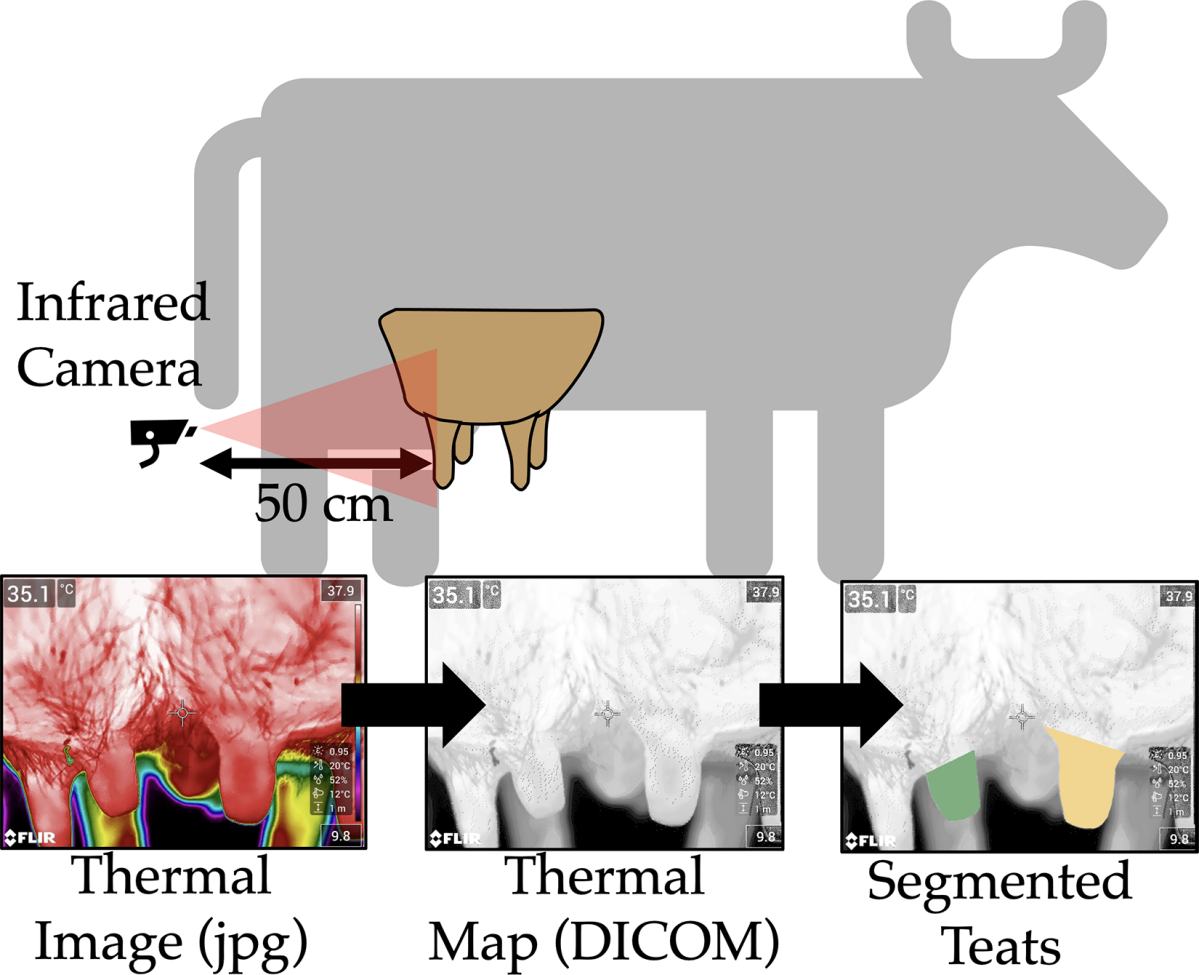
A thermal radiomics workflow for analyzing dairy cow teats. The digital thermography camera is positioned 50 cm from the hind teats and a thermal image is acquired. The thermal image is then converted from a 3-channel colorized image (jpg) to a single-channel Digital Image and Communications in Medicine (DICOM) image data format. Then, an open-source medical image software package is used to manually segment the left (green) and right (yellow) hind teats.

In this article, we describe a novel approach for quantitatively detecting postmilking changes in the surface temperature of teats of dairy cows through radiomics analysis of thermal images. First, we articulate the application of radiomics methodology to thermal imaging. Second, we describe the manual segmentation of teats and the inter- and intraobserver segmentation variabilities and their effect on radiomics calculations. Finally, we illustrate how thermal radiomic feature differences can be used to detect changes in teats and how nonsemantic temperature features of the teat may be appreciated.

The study protocol was reviewed and approved by the Cornell University Institutional Animal Care and Use Committee (protocol no. 2021-0005). The methodology for this analysis is shown in Figure 1. Thermographic images of both hind teats were obtained in the milking parlor from the caudal aspect of the udder in a caudo-to-cranial direction from a distance of approximately 50 cm with a portable thermographic camera (FLIR T530, Teledyne FLIR LLC). The fixed distance ensured that temperature measurements were consistent and teat dimensions were comparable when imaged at the highest resolution (640 width × 480 height). Before the study, the imaging modes were identified and kept consistent throughout the trial. We used the autofocus function “laser” option, where the focus is based on a laser distance. The laser distance meter was enabled to automatically determine the object's distance. We used a fast shutter speed and manual repositioning of the camera to reduce the risk of motion artifacts. The atmospheric temperature and relative humidity retrieved from the local weather station were 23°C and 81%, respectively. The reflective temperature was kept at 20°C and emissivity at 0.95. All scans were labeled with the cow identification number and time relative to milking (before and after milking) and subsequently stored on the integrated flash drive in lossless JPG format. The saved data consisted of a colorized image along with a colormap that related the color to the temperature embedded within the image along with the scale (maximum and minimum temperatures). Images were then exported to a computer for image analysis.

For thermographic images to be quantitatively analyzed, the colorized image must be converted to an image where pixel intensities represent temperatures in the image. This was achieved by using a custom program written in Matlab (version 2021a, Mathworks). First, the image was read by software and the range of temperatures observed in the current image (displayed in the colorized JPG image on the right-hand side alongside the colorbar) was manually entered in the program. A lookup table was created that converts a 3-channel colormap of the colorized image to a single-channel grayscale image. The dynamic range of the colorized image is no greater than 256 × 3 channels, or 16,777,216 unique colors. The temperature of the teats and background range from approximately 5 to 40°C. Because the device has a precision of 0.1°C, no more than ~350 unique temperatures can be discretized from the image. Thus, little to no interpolation of temperatures is necessary to covert a unique color to temperature with a precision of 0.1°C. Although the temperature map could be saved in 8-bit floating point precision for quantitative analysis, the temperature intensities were scaled up by a factor of 10 and converted to unsigned integers to (1) match formats commonly observed in medical image analysis visualization software; and (2) truncate the precision in the temperature values such that a precision of no greater than 0.1°C is reflected in the subsequent analysis. Images are saved in standard DICOM (Digital Image and Communications in Medicine) medical image format.

Using a medical imaging format for the thermographs allowed us to leverage the immense number of open source and downloadable software packages and products available for quantitative medical image analysis. The freely available 3DSlicer package was used to import the medical images, manually segment the left and right hind teats, and perform quantitative analysis of the segmented image (3D Slicer; https://www.slicer.org/, accessed March 15, 2021). Segmentation refers to the identification and delineation of a region of interest and assigning it a label, such as “left hind teat” or “right hind teat.” For the present radiomics analysis, 2 regions of interest were defined for analysis: the left and right hind teats. The segmentation was achieved by identifying the perimeter of the teat, as projected in the image using the “Draw” tool within 3DSlicer. Radiomics calculations were then performed over the segmented region of interest. The distal aspects of the teat can be easily identified in the image due to the large temperature differences between the teat and the background; however, determining the proximal extent of the teat may be prone to differing interpretations because the shape and boundary delineating the teat from the udder can vary within and between cows. We standardized the segmentation of the teat by tracing the outer perimeter of the teat and used a tangent line to close the segment where the teat appeared to attach to the udder as projected in the image.

Once the teats were segmented on the thermographs, image biomarkers were computed. The “Pyradiomics” package within 3DSlicer consists of a suite of programs that can compute up to 851 image biomarkers from a region of interest within a 2D or 3D image. Radiomics analysis provides quantitative morphometric, first-order intensity, and higher-order texture differences. The image spatial dimensions are not calibrated in an absolute sense. Therefore, features that describe the teat shape, such as width, length, and surface area, may not be suitable metrics for detecting image biomarkers. However, other shape features that examine the ratios of length and width, such as eccentricity and sphericity of the teat, do not rely on an absolute distance measure. The first-order texture features are of greater interest. They provide metrics such as the mean, median, range, skewness, and kurtosis of temperature values in the region of interest. Second- and higher-order features provide insights on how temperature varies across the segmented image of the thermogram. Texture features are computed by representing how pixels of temperature in the left or right hind teats are spatially arranged. This is done by collapsing the temperature distribution in the segmented teat into a matrix. Some important texture matrices are the gray-level co-occurrence matrix (**GLCM**), which characterizes the distribution of co-occurring temperature pixels horizontally, vertically, or diagonally within the segmented teat; the gray-level dependence matrix (**GLDM**), which assesses the number of similar and connected temperature pixels relative to the temperature in the center of the teat; and the neighborhood gray tone distance matrix (**NGTDM**), an alternative to the GLCM, where the sum of temperature differences is computed, rather than the temperature of the pixels inside the teat. More details of these matrices and their corresponding image biomarkers may be found elsewhere (van Griethuysen et al., 2017; Basran et al., 2021).

We demonstrated the utility and potential of thermal radiomics with thermal images obtained from healthy dairy cows (50 teats). To examine uncertainties introduced from segmentation on thermal radiomic features, we manually segmented and computed features of a convenience sample from 25 thermal images of hind teats. To examine whether changes in thermal radiomic features were detectable, 18 healthy dairy cows (36 teats) were scanned before and 60 s after milking, teats were manually segmented, and features were computed. All images were converted to thermal DICOM images, and 3DSlicer was used to segment the 2 hind teats by 2 investigators (MW and PB). Radiomic calculation settings from the Pyradiomics package within 3DSlicer were as follows: no image interpolation, fixed bin width = 0.1°C, range re-segmentation = “off,” symmetrical GLCM = “on,” no LoG kernel smoothing was applied, and all wavelet-based features computed (851 image biomarkers computed in total per teat). Data were exported in comma delimited format for statistical analysis using Matlab and Excel (Microsoft Corp.).

Uncertainties from segmentations were examined by comparing segmentations from the 2 investigators. Dice similarity scores (**DS**) were computed for each of the 100 teats segmented and compared, where a value of 1.00 represents a perfect overlap of the segments, and 0.50 represents a 50% (area) overlap. When examining both left and right hind teats, the average DS and 95% confidence limit was 0.95 (0.91–0.98). We detected no meaningful difference in DS between the left (0.96, 0.92–0.98) and right (0.95, 0.88–0.98) segmented teats, as indicated by the overlapping confidence limits. This suggests that the teats can be segmented consistently by the 2 investigators. No differences (*P* > 0.07, Cohen's *d* < 0.36) were observed when comparing image biomarkers based on one investigator's segments versus the other's (Cumming, 2014). This suggests that the image biomarkers computed from one investigator's segmentation of teats are not likely to differ from those computed from the other investigator. In other words, there is consistency in the computed image biomarkers extracted from the segmentations generated by the 2 investigators.

One investigator segmented the hind teats of 18 dairy cows from which thermal images were collected before and after milking. We hypothesized that the temperature of the teat ends increases after milking. Student's *t*-test (*P* = 0.01) was used to estimate the likelihood that the postmilking feature estimate is the same as the premilking feature. Because statistical difference does not provide insight on the size of an effect, we used Cohen's *d* to evaluate the size of the effect (*d* > 1.2) for image biomarkers before and after milking (Cumming, 2014). Only 109 of 851 image biomarkers are reported here: all 851 feature calculations from the 50 segmented teats along with code used in this work are available online (https://github.com/pbasran/delta-thermal-radiomics). We defined an image biomarker as expressed when *P* < 0.01 and *d* > 1.2.

When comparing biomarker differences from pre- and postmilking, 21 of the 109 image biomarkers were statistically different, and 19 exhibited effect size. Of these expressed image biomarkers, 17 image biomarkers were simultaneously significant and exhibited effect size. This observation, along with our previous findings of high DS when segmenting the teats, suggests that not only are there are substantial numbers of expressed image biomarkers, but also that image biomarkers are not sensitive to uncertainties in segmentation between the 2 investigators. Some common first-order image biomarkers included 10th and 90th temperature histogram percentiles entropy, mean, median, root mean square, and autocorrelation (Table 1). Some of the second-order image biomarkers included GLCM-Cluster shade, which is a measure of the skewness and uniformity; GLCM-Sum average, which is a measure of the relationship between occurrences of pairs with lower temperature pixels and occurrences of higher temperature pixels; GLDM-High gray-level emphasis, which is a measure of the concentration of higher temperature pixels; and NGTDM-Strength, which measures the number of distinguishable shapes in the image. The table also displays biomarker estimates for GLDM- large dependence high gray-level dependence emphasis, which is a much more complex feature that measures the joint distribution of large dependence with higher temperature pixels.

**Table 1:** Changes in select thermal radiomic features before and after milking^1^

Feature name^2^	Before Mean (95% CI)	After Mean (95% CI)	Absolute difference in mean, %
Mean	31.2 (29.1, 32.1)	31.3 (29.4, 32.9)	7.3
Median	32.2 (29.1, 33.5)	33.9 (32.1 35.6)	8.7
10th percentile histogram	29.5 (27.0, 32.1)	31.3 (29.4, 32.9)	5.9
90th percentile histogram	33.0 (30.8, 34.9)	35.1 (33.4, 36.5)	6.4
Root mean square	31.3 (29.1, 33.4)	33.5 (32.0, 34.9)	7.3
Autocorrelation	1.58 (4.54, 31.27) Ã— 10^4^	3.56 (1.32, 5.85) Ã— 10^4^	125.0
GLCM-Cluster shade	19.0 (−22.3, 28.6) Ã— 10^4^	−32.3 (−64.2, −42.7) Ã—10^4^	1,793
GLCM-Sum average	231 (126, 349)	360 (224, 479)	55.6
GLDM-High gray-level emphasis	1.59 (0.47, 3.13) Ã— 10^4^	3.56 (1.32, 5.82) Ã— 10^4^	123.5
GLDM-Large dependence high gray-level dependence emphasis	4.59 (1.21, 11.50) Ã— 10^5^	11.23 (2.68, 22.24) Ã— 10^5^	145.1
NGTDM-Strength	13.7 (4.6, 21.2)	24.5 (11.6, 54.9)	83.1

1 All features here have *P* < 0.01 and Cohen's *d* >1.2 (i.e., demonstrate a significant effect size). All numbers are reported with 4 or fewer significant digits.

2 GLCM = gray-level co-occurrence matrix; GLDM = gray-level dependence matrix; NGTDM = neighborhood gray tone distance matrix.

Figure 2 displays expressed feature maps for the mean temperature, skewness, temperature gradients, and entropy of a teat pre- and postmilking, computed over a 10 × 10 pixel window. The mean temperature of teats was found to increase after milking. In addition to bulk changes in temperature, the feature map provides a distribution of temperature changes pre- and postmilking over the skin surface. Note how the teat-end temperature is relatively the same before and after milking. Figure 2b displays changes in temperature skewness over the 10 × 10 window, which demonstrated a large effect size and significant difference before and after milking. Figure 2c illustrates the 2-dimensional temperature gradient, showing the boundaries of isotherms, and Figure 2d displays the entropy scores over the entire teat. Although the average temperature change may be a useful metric in estimating blood flow and other changes, other metrics revealed through thermal radiomics may detect how and where skin temperatures change before and after milking. Moreover, several of these features (e.g., skewness, GLCM inverse difference moment) are independent of mean temperature and can offer parameters for modeling short-term teat physiological changes associated with machine milking. Figure 2b illustrates the postmilking reduction in histogram skewness and GLCM-Cluster shade, which is a similar measure of the skewness and uniformity to the gray-level co-occurrence matrix.

**Figure 2:**
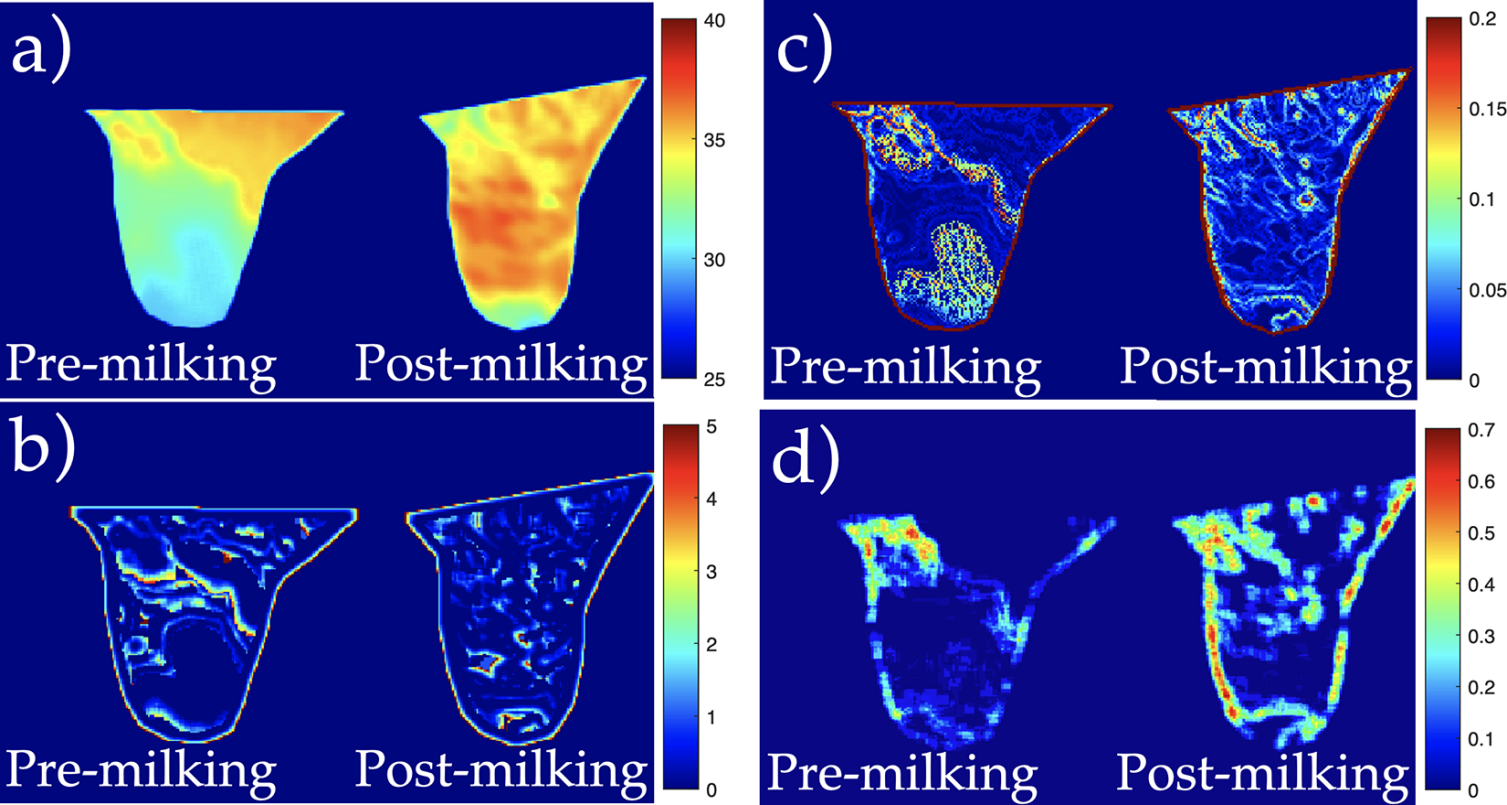
Pre- and postmilking radiomic differences illustrated through feature maps. The subpanels display feature maps computed over 10 × 10 pixels for (a) mean temperature, (b) first-order histogram skewness, (c) temperature gradient, and (d) entropy. Panel (a) suggests the central regions of the teat exhibit most of the temperature change after milking. Panel (b) suggests that the overall distribution of temperature histograms is shifted toward cooler temperatures before milking and toward hotter temperatures after milking, which is independent of the mean temperature difference (Pearson correlation = 0.072). Panel (c) suggests that temperature gradients at the teat-end are larger before milking than after milking, even though the mean temperature of the teats increases after milking. Panel (d) suggests a greater variation in temperatures after milking compared with variations before milking.

Interestingly, although differences in morphological image biomarkers from pre- and postmilking images were not anticipated, some differences were identified. The major axis length, which corresponds to the long axis of the teat, increased by 31% after milking, and the surface area of the segmented teat increased by 50%. Although this radiomic feature is not the same as the 3-dimensional teat surface area, it does provide a surrogate measurement of area as seen from the camera. This observation is consistent with results from previous studies (Guarín and Ruegg, 2016; Melvin et al., 2019) and is most likely attributable to longitudinal shear forces that are applied to the teat during milking (Williams and Mein, 1982). Elongation of teats after milking was higher and did not reach our level of significance (*P* < 0.03) but it demonstrated a substantial effect size (*d* > 1.20), suggesting that detection of morphological changes via thermal radiomics is plausible. However, care is required in interpreting this result because the pixel intensities are not spatially calibrated. In delta radiomics from medical images, changes in morphometric image biomarkers can be quantified on an absolute scale because the imaging system and the corresponding image are calibrated; this is not the case with our thermal images. One possible ameliorative approach is to calibrate the pixel sizes through dual-camera infrared surface imaging, commonly used in the gaming and 3D printing communities (Zhang, 2000; Geng, 2011).

Some additional challenges with thermal radiomics include the temperature resolution of the thermal imaging system, currently limited to 0.1°C with our camera, noise in the thermal images, and possible interpolation errors when migrating colorized images to grayscale thermal temperature images. Although there may be subtle temperature features that could be detected if the temperature resolution was finer (e.g., 0.01°C discretization), the reliability and clinical relevance of increasing the precision in these measurements (e.g., by a factor of 10) remains unclear. Impulse noise, often referred to as “salt and pepper” noise in the image, is often observed in thermal images. This may be due to inaccurate temperature readings from the thermograph and interpolation errors when mapping colorized images to thermal maps. The former may be addressed by additional environmental controls during imaging, such as conducting measurements indoors under climate and dust control, whereas the latter may be addressed by accessing the raw temperature map readings from the camera itself and bypassing the generation of a colorized thermal image. Post-processing of the original or converted thermal images is also possible, including wavelet transforms and Laplacian of Gaussian filtering, which are readily deployable within the Pyradiomics environment (Skogen et al., 2016). Whether the uncertainties in image biomarkers are comparable to the uncertainties in manual thermographic imaging is a subject of future investigation.

Several interesting opportunities are presented with a delta thermal radiomics approach, particularly within the scope of digital agriculture and machine learning. By using a thermal radiomics approach, it may be possible to quantify the extent of skin temperature alterations more precisely. If an association between skin temperature and physiological variables such as blood flow is established, thermal radiomics image maps may be useful in delineating sub-regions of surface blood-flow variations as a function of time after an intervention, such as milking. In addition to characterizing temperature changes in a quantitative and spatial manner over the short term (e.g., before and after milking), thermal radiomics may be performed over longer time intervals, such as over the course of weeks, for monitoring long-term changes to the teat. Given the immense number of image biomarkers extractable from a radiomics approach, application of feature selection methods common in the machine learning and data sciences becomes necessary. However, the most time-expensive process in a thermal radiomics workflow is the manual segmentation of teats for analysis. The advent of U-Nets (neural network) and higher processing speeds have rendered auto-segmentation a relatively straightforward and simple task (Huang et al., 2017). Once the teats are segmented, features can be computed and used for monitoring thermally regulated phenomena. When using a standardized approach for segmenting the teats, the thermal radiomic image biomarkers do not appear to be sensitive to interobserver segmentation uncertainties.

To conclude, we describe a novel approach for analyzing thermal images by way of radiomics. The expressed thermal image biomarkers of the teat skin could reveal insights into the underlying changes in blood flow caused by milking. This thermal radiomics approach can detect both perceptible and imperceptible features that could be used for detecting and managing dairy teat health. A delta thermal radiomics approach offers a noninvasive and quantitative method of monitoring changes in skin temperature in animals—and humans—with software that is readily accessible and well documented.
